# Chk1 Inhibition Ameliorates Alzheimer’s Disease Pathogenesis and Cognitive Dysfunction Through CIP2A/PP2A Signaling

**DOI:** 10.1007/s13311-022-01204-z

**Published:** 2022-03-14

**Authors:** Wenting Hu, Zhuoqun Wang, Huiliang Zhang, Yacoubou Abdoul Razak Mahaman, Fang Huang, Dongli Meng, Ying Zhou, Shiyi Wang, Nan Jiang, Jing Xiong, Jukka Westermarck, Youming Lu, Jianzhi Wang, Xiaochuan Wang, Yangping Shentu, Rong Liu

**Affiliations:** 1 Department of Pathophysiology, Key Laboratory of Ministry of Education for Neurological Disorders, School of Basic Medicine, Tongji Medical College, Huazhong University of Science and Technology, Wuhan, China; 2 Cognitive Impairment Ward of Neurology Department, The Third Affiliated Hospital of Shenzhen University, Shenzhen, China; 3 Department of Nephrology, The First Affiliated Hospital of Wenzhou Medical University, Wenzhou, China; 4 Wenzhou Medical University, Wenzhou, China; 5 Department of Pathology, The First Affiliated Hospital of Wenzhou Medical University, Wenzhou, China; 6 Department of Nephrology, Union Hospital, Tongji Medical College, Huazhong University of Science and Technology, Wuhan, China; 7 Turku Centre for Biotechnology, University of Turku and Abo Akademi University, Turku, Finland; 8 Institute of Biomedicine, University of Turku, Turku, Finland; 9 Collaborative Innovation Center for Brain Science, The Institute of Brain Research, Huazhong University of Science and Technology, Wuhan, China

**Keywords:** Alzheimer’s disease, Chk1, CIP2A, PP2A, Tau, APP, Hyperphosphorylation

## Abstract

Alzheimer’s disease (AD) is the most common neurodegenerative disease with limited therapeutic strategies. Cell cycle checkpoint protein kinase 1 (Chk1) is a Ser/Thr protein kinase which is activated in response to DNA damage, the latter which is an early event in AD. However, whether DNA damage-induced Chk1 activation participates in the development of AD and Chk1 inhibition ameliorates AD-like pathogenesis remain unclarified. Here, we demonstrate that Chk1 activity and the levels of protein phosphatase 2A (PP2A) inhibitory protein CIP2A are elevated in AD human brains, APP/PS1 transgenic mice, and primary neurons with Aβ treatment. Chk1 overexpression induces CIP2A upregulation, PP2A inhibition, tau and APP hyperphosphorylation, synaptic impairments, and cognitive memory deficit in mice. Moreover, Chk1 inhibitor (GDC0575) effectively increases PP2A activity, decreases tau phosphorylation, and inhibits Aβ overproduction in AD cell models. GDC0575 also reverses AD-like cognitive deficits and prevents neuron loss and synaptic impairments in APP/PS1 mice. In conclusion, our study uncovers a mechanism by which DNA damage-induced Chk1 activation promotes CIP2A-mediated tau and APP hyperphosphorylation and cognitive dysfunction in Alzheimer’s disease and highlights the therapeutic potential of Chk1 inhibitors in AD.

**Supplementary Information:**

The online version contains supplementary material available at 10.1007/s13311-022-01204-z.

## Introduction

Alzheimer’s disease (AD) is a common neurodegenerative disorder accompanied by progressive development of cognitive impairments and synaptic dysfunction. With the aging of the global population, the number of AD patients has increased sharply, which has caused a huge economic burden on society and families. The two characteristic pathological hallmarks in AD are the formation of neurofibrillary tangles (NFTs) and senile plaques (SPs) in the brain [1, 2]. The major components of NFTs and SPs are hyperphosphorylated tau and Aβ, both of which have toxic effects on synaptic function and induce the damage and loss of neurons in the AD brain.

Cancerous inhibitor of PP2A (CIP2A) is an endogenous inhibitor of protein phosphatase 2A (PP2A). CIP2A is an oncoprotein proved to be upregulated in a variety of peripheral tumors, and promotes tumor cell growth through inhibiting the dephosphorylation of PP2A substrates which are involved in cancer development [3, 4]. We and others showed that CIP2A was also expressed in the brain of humans and mice. Our previous research indicated that in the brain of AD human and transgenic mice, the expression of CIP2A increased and overexpression of CIP2A induced tau and APP hyperphosphorylation through inhibiting PP2A, causing cognitive and memory impairment and synaptic dysfunction [5]. Furthermore, CIP2A is also upregulated in AD astrocytes. Specific overexpression of CIP2A in mouse brain astrocytes resulted in reactive astrogliosis, which promote synaptic degeneration and cognitive deficits [6]. Thus, CIP2A is a key disease-promoting factor in AD. However, the mechanism of CIP2A upregulation in AD remains unclear.

Cell cycle checkpoint kinase 1 (Chk1) is a Ser/Thr protein kinase which plays an important role in DNA damage response (DDR). As a key DNA damage checkpoint kinase, Chk1 is phosphorylated and activated by DNA damage sensor kinases ataxia-telangiectasia mutated (ATM), ataxia-telangiectasia mutated and rad3 related (ATR), and DNA-dependent protein kinase (DNA-PK) [7, 8]. In proliferating cells, activated Chk1 can phosphorylate and inactivate cdc25C and cdc25A, preventing their effects of removing the inactivating phosphate on CDK1 and CDK2, thus inhibiting G2/M progression and S-phase entry. In addition, Chk1 also participates in DNA repair through multiple signaling pathways [9, 10]. Despite the thorough investigation of Chk1’s function in dividing cells, its precise role in differentiated cells such as neurons in the mature brain has not been characterized extensively.

Neuronal DNA damage is a hallmark of AD brains [11, 12]. A recent study furthermore demonstrated double-strand breaks (DSBs) both in neurons and astrocytes in the hippocampus and frontal cortex of mild cognitive impairment (MCI) and AD patients, indicating that DNA damage is an early event in AD development [13]. On the other side, it was previously demonstrated that, in addition to its classical role in DNA damage checkpoint regulation, Chk1 activity in tumor cells feeds to cellular phosphoproteome regulation by driving the expression of protein phosphatase 2A inhibitor protein CIP2A [8]. More recently, it was shown that Chk1 constitutively phosphorylated on serine 345 promoted CIP2A expression via transcriptional mechanisms involving STAT3 [14]. However, whether the Chk1-CIP2A-PP2A signaling axis is functionally relevant outside cancer is currently unknown. In the present study, we reveal the increased DNA damage accompanied with Chk1 activation in AD human brains and animal and cell models, and demonstrate that neuronal DNA damage-induced Chk1 activation promotes the AD-like pathology by CIP2A upregulation. We also provide a preclinical rationale for testing Chk1 inhibitors as novel AD therapeutics.

## Materials and Methods

### Antibodies and Reagents

The details of primary antibodies used in this study are all shown in Table 1. The second antibodies were obtained from LICOR Biosciences (Cat#C50133-06 and Cat#C50331-05). AAV virus was constructed and packaged by Genechem (Shanghai, China). GDC-0575 (Chk1 inhibitor), PEG300, and Tween-80 were purchased from MedChemExpress (New Jersey, USA).

**Table 1:** Information of the primary antibodies

Antibodies	Epitopes	Type	Dilution	Sources
Chk1 Chk1 Chk1 Chk1-S296 Chk1-S317 Chk1-S345 Chk1-S345 CIP2A γH2A.X 53BP1 S199 S396 S404 AT8 Tau-5 APP T668 SYP Synapsin I GluA1 PSD95 GAPDH Actin	Chk1 Chk1 Chk1 Chk1 (Ser296) Chk1 (Ser317) Chk1 (Ser345) Chk1 (Ser345) CIP2A Gamma H2A.X (Ser139) 53BP1 Tau (Ser199) Tau (Ser396) Tau (Ser404) Tau (Ser202/Thr205) total tau APP APP (Thr668) Synaptophysin Synapsin I GluA1 PSD95 GAPDH Actin	pAb pAb pAb mAb mAb mAb mAb pAb mAb pAb pAb pAb pAb pAb mAb mAb mAb mAb pAb mAb mAb mAb mAb	1/1000 1/1000 1/1000 1/500 1/500 1/500 1/1000 1/1000 1/1000 1/1000 1/1000 1/1000 1/1000 1/1000 1/1000 1/1000 1/1000 1/1000 1/1000 1/1000 1/1000 1/1000 1/1000	Abclonal (Cat# A7653) zen-bioscience (Cat# 380,200) bioss (Cat# bs-1681R) Abclonal (Cat# AP1047) Cell Signaling (Cat# 12,302) Gene Tex (Cat# 39,233) Cell Signaling (Cat# 2348) Abclonal (Cat# A12267) Abcam (Cat# ab26350) zen-bioscience (Cat# 381,816) zen-bioscience (Cat# 383,320) SAB (Cat# #11,102) SAB (Cat# #11,112) zen-bioscience (Cat# 382,945) Abcam (Cat# ab80579) Proteintech (Cat# 60,342–1-Ig) Cell Signaling (Cat# 6986) Santa Cruz Biotechnology (Cat# sc-17750) absin (Cat# abs124586) Cell Signaling (Cat# 13,185) merck millipore (Cat# MABN68) Proteintech (Cat# 66,004–1-Ig) Proteintech (Cat# 66,009–1-Ig)

### Human Brain Samples and Mice

Human brain samples were from the China Brain Bank (Zhejiang University School of Medicine). More detailed information is in Table 2. C57B6/L mice were obtained from the Experimental Animal Center of Tongji Medical College, Huazhong University of Science and Technology. C57/BL6 mice aged 8 weeks were injected with the virus. One month later, the behaviors of the mice were tested, which lasted for 2–3 weeks. Finally, the mice were sacrificed for molecular biochemical assay at the age of 14–15 weeks. APP/PS1 mice were from Jackson Lab. These mice are double transgenic mice expressing a chimeric mouse/human amyloid precursor protein (Mo/HuAPP695swe) and a mutant human presenilin 1 (PS1-dE9), both directed to CNS neurons. In our experiment, APP/PS1 mice aged 8 months were treated with GDC-0575 for 3 weeks, followed by behavioral tests for 2–3 weeks. Finally, the APP/PS1 mice were sacrificed for molecular biochemical assay at the age of 10 months. During the experiments, all animals were kept in a condition with appropriate temperature (22 ± 2 °C), humidity (55 ± 15%), and 12–12 h light–dark cycle. All animal experiments were approved by the Animal Care and Use Committee of Huazhong University of Science and Technology and performed in compliance with the NIH Guide for the Care and Use of Laboratory Animals.

**Table 2:** Information of human samples

Sample	Age	Sex	Braak stage	Brain region	Genotype
CTR-1	72	Male	N/A	Hippocampus	N/A
CTR-2	83	Male	N/A	Hippocampus	N/A
CTR-3	86	Male	N/A	Hippocampus	N/A
CTR-4	98	Female	N/A	Hippocampus	N/A
CTR-5	83	Female	N/A	Hippocampus	N/A
AD-1	78	Male	4	Hippocampus	N/A
AD-2	71	Female	4	Hippocampus	N/A
AD-3	73	Male	4	Hippocampus	N/A
AD-4	79	Male	6	Hippocampus	N/A
AD-5	99	Female	5	Hippocampus	N/A

### Cell Culture and Treatment

For HEK293/Tau and N2a/APP cell culture, cells were cultured in DMEM-high glucose medium supplemented with 10% fetal bovine serum in an incubator of 5% CO_2_ at 37 °C. When cells reached 70–80% confluence in six-well plates, they were treated with GDC-0575 at different concentrations for 24 h. Then, cells and culture media were collected for further experiments.

### Primary Neuron Culture and Treatment

Primary cortical neurons were prepared from E16-E18 Sprague–Dawley rat embryos. Tissues were dissected and digested by trypsin for 20 min, followed by the addition of the neuronal plating medium containing DMEM/F12 with 10% fetal bovine serum to terminate the digestion. Then, the cell suspension was seeded onto plates coated with poly-D-lysine and incubated in an incubator at 37 ℃ in the presence of 5% CO_2_. After 4–6 h, medium was replaced with neurobasal medium supplemented with 2% B-27, 1% GlutaMAX (2 mM), 1% penicillin (50 U/mL), and streptomycin (50 μg/mL). Neurons were cultured for 9 days before treatments and the medium was half-changed every 3 days with fresh maintenance media during the culture. For Aβ treatment, human Aβ_42_ peptides (chinapeptides, Shanghai, China) were dissolved in DMEM-high medium at a concentration of 200 μM and incubated at 4 ℃ for 14–16 h for oligomer formation before use. The Aβ oligomers were added to the cell medium at a final concentration of 2 μM for 48 h. For Chk1 inhibition by SB218078 and PF477736, the neurons were incubated with SB218078 or PF477736 (dissolved in DMSO) at a concentration of 1 μM for 48 h. For H_2_O_2_ treatment, primary neurons were cultured with 200 µM H_2_O_2_ for 2 h with or without pre-incubation of Chk1 inhibitor SB218078 (1 μM) or PF477736 (1 μM) for 48 h before collecting cells.

### RNA Extraction, Reverse Transcription, Quantitative Real-Time Polymerase Chain Reaction

RNA was extracted using Trizol Reagent (Invitrogen, Waltham, MA, USA). For details, 1 ml Trizol reagent was added into each well and mixed thoroughly, followed with cooling on ice for 5 min, spinning at 12,000 g, 4 °C for 10 min. The supernatants were removed into new tubes, vortex mixed with 500 μl chloroform for 15 s. The mixture was incubated on ice for 5 min, and centrifuged at 12,000 g, 4 °C for 10 min. Next, the top layer of the solution was carefully transferred into new tubes, and 1:1 V of isopropanol was added to each tube, mixed by inversion, and centrifuged at 12,000 g for 10 min at 4 °C. The supernatant was discarded, and the pellet was washed with 1 ml 75% ethanol, centrifuged at 7500 g for 5 min. Removed all of the residual ethanol and dried the samples in air for 3 min. RNA was re-dissolved in 30 μl DEPC H_2_O (diethyl pyrocarbonate H_2_O). Total RNA concentration was measured (Synergy H1, BioTek, USA) and the concentration of all samples was corrected to 500 ng/μl. Next, first-strand complementary DNA (cDNA) was synthesized from 500 ng total RNA using the high-capacity cDNA reverse transcription kit (First-Strand cDNA Synthesis Kit, TOYOBO, China). Quantitative polymerase chain reaction (PCR) was performed in a 20 μl standard PCR reaction mixture in accordance with the manufacturer’s protocol (Hifair® III One Step RT-qPCR SYBR Green Kit, Yeasen Biotech, Shanghai, China, Cat# 11143ES50). Quantitative PCR primers were as follows: CIP2A, sense: 5’-gaacagataagaaaagagttgacatt-3’ and antisense: 5’-gaacagataagaaaagagttgagcatt -3’. The actin primers were used as the internal control. Actin, sense: 5’-cacagactacctcatgaagatcc-3’ and antisense: 5’-cagctcgtaactcttctccag -3’.

### Stereotaxic Injection

C57B6/L mice were injected with the AAV virus stereotaxically under anesthesia. pAAV9-SYN-Chk1-EGFP-3FLAG and control vector were from Genechem (Shanghai, China). Bilateral ventricles stereotaxic injections (anteroposterior, − 0.2 mm; mediolateral, ± 0.9 mm; dorsoventral, − 2.3 mm from bregma) of 2 µl high-titer AAV (~ 10 ^12^ GU/ml) were performed with a Hamilton syringe at a rate of 0.2 µl/min. One month later, mice were tested regarding the behaviors and then sacrificed for further detections.

### Drug Administration

The mice were divided into three groups: control group (C57B6/L mice), AD group (APP/PS1 mice aged 8 months), and treatment group (APP/PS1 mice aged 8 months administrated with GDC-0575). GDC-0575 was solubilized in 10% DMSO (25 mg/ml), 40% PEG300, 5% Tween-80, and 45% saline and mixed to obtain a final concentration of 3.75 mg/ml. Mice of the treatment group were treated with GDC-0575 (25 mg/kg) in 2 of 7 days by oral gavage. Others were administrated with the corresponding solvent. The whole experiment lasted for 3 weeks.

### Western Blotting

Cell and brain tissue were lysed with RIPA lysis buffer (Beyotime Biotechnology, Shanghai, China) containing PMSF (1:100) and proteinase inhibitor cocktail (200 mM AEBSF, 30 μM aprotinin, 13 mM bestatin, 1.4 mM E64, and 1 mM leupeptin in DMSO, 1:100) (Yeasen Biotech, Shanghai, China Cat#20,124), then boiled for 10 min. The brain homogenates and cell lysis were centrifuged for 10 min at 12,000 g at 4 °C, followed by sonication and determination of protein concentration by BCA kit (Thermo Fisher Scientific, Waltham, MA, USA).

The extracted proteins were resolved by 10% SDS–polyacrylamide gel then transferred onto nitrocellulose membranes (Amersham Biosciences, USA). Then, the membranes were blocked with 5% non-fat milk for 1 h and incubated overnight at 4 °C with primary antibodies. The membranes were washed by TBST buffer 3 times for 10 min each and incubated with 1:10,000 anti-mouse or anti-rabbit secondary antibody for 1 h at room temperature followed by visualization using Odyssey Infrared Imaging System (LICOR Biosciences, USA).

### LDH Cytotoxicity Assay

The LDH cytotoxicity assay was executed according to the manufacturer’s procedure (Cat# C0017, Beyotime Biotechnology, Shanghai, China).

### Aβ_40/42_ Assay by ELISA

The supernatants of N2a/APP cells were collected for detection of Aβ_40/42_ levels. For cell lysate, N2a/APP cells were lysed in PBS (containing 1:100 PMSF and 1:100 protease inhibitor cocktail); then, samples were centrifuged for 5 min at 3000 g at 4 ℃ and crushed by ultrasound. The supernatants were collected for detecting Aβ_40/42_ levels. For brain tissue, the hippocampus was homogenized with PBS (containing 1:100 PMSF and 1:100 protease inhibitor cocktail) and centrifuged for 10 min at 12,000 g at 4 ℃. The supernatant was collected as the RIPA‐soluble fraction. The pellets were dissolved in 70% formic acid as the RIPA-insoluble fraction. The Aβ_40/42_ levels of RIPA-soluble or insoluble fraction were detected according to the manufacturer’s instructions (Elabscience Biotechnology, Wuhan, China).

### PP2A Activity Assay

Supernatants of the HEK293/tau, N2a/APP cell lysates, and mice brain tissue extracts were prepared. PP2A activity in the supernatants was assayed using the Serine/Threonine Phosphatase Assay kit V2460 according to the manufacturer’s procedure (Promega, Madison, USA). Briefly, endogenous free phosphate was removed from supernatants, and then the extracts were normalized for protein content, 5 μg protein samples in triplicates were incubated with a chemically synthesized phosphopeptide (RRA(pT)VA), an optimal substrate for PP2A, PP2B, and PP2C, but not for PP-1 in a buffer optimized for PP2A activity while cation-dependent PP2B and PP2C were inhibited for 30 min at 33 °C [15]. Phosphate release from the substrate was detected by measuring the absorbance of a molybdate-malachite green-phosphate complex at 630 nm. The activity of PP2A was evaluated by the release of phosphate per μg protein and per minute (pmol/μg/min).

### Chk1 Activity Assay

Chk1 activity was performed using the kinase assay kit according to the protocol (Cat# GMS50155.2, GENMED SCIENTIFICS INC, Boston, USA).

### Fluorescence Microscopy

The mice were anesthetized by isoflurane and perfused intracardially with normal saline until the liver and the spleen turned grayish-white; then, 30–40 ml of 4% PFA was perfused to fix the brain tissue. After perfusion, the brain was removed; hemispheres of the mice were further fixed with 4% PFA at 4 °C for 16 h, then washed in phosphate buffer (PB, 0.1 M, pH 7.4), immersed in 30% sucrose in PB till the tissue blocks sink to the bottom. Then, the fixed brains were embedded in OCT, frozen, and coronally sectioned at 30 μm using a cryotome (CM1950, Leica, Germany). Brain slices were washed with PBS three times and incubated with Hoechst (1:3000) for 7 min to stain nuclei. After wash, brain slices were covered with glass coverslips and observed under a fluorescence microscope (LSM710, Zeiss, Germany).

### Behavior Tests

#### Open Field Test

The mice were arranged in an empty area (50 cm × 50 cm × 50 cm plastic container, Techman Software Co., Ltd., Chengdu, China) for 5 min. The region was equally divided into 5 × 5 sectors, and the middle 3 × 3 sectors were defined as the center area. The time and distance of moving were recorded.

#### Novel Objective Recognition Test (NOR)

The mice were arranged in an area (50 cm × 50 cm × 50 cm plastic container) for 5 min without any other object 24 h before the experiment begins. On the first day of testing, the mice re-entered the area with object A and object B (the shape and color of object A and object B are different) from the same point, and were granted to be familiar with object A and object B for 5 min; the area was cleaned with 75% ethanol between each habituation period. After 24 h, the object B was replaced by object C (the shape and color of object A and object B and object C are different); same as before, the mice re-entered the area with object A and object C from the same point; the exploring time of the mice on object A and C was recorded. We recorded the exploration of the mice on the two objects, including the number of times the nose or mouth touched the object and the exploration time within 2–3 cm of the object (front paw on the object, nose sniffing the object, licking objects, etc.). The recognition index was calculated as described below. The first day is TA/(TA + TB), TB/(TA + TB), the second day is TA/(TA + TC), TC/(TA + TC). TA, TB, and TC represent the exploring time of the mice on objects A, B, and C respectively.

#### Morris Water Maze Test (MWM)

Spatial learning and memory were detected by the Morris water maze (MWM, Techman Software Co., Ltd., Chengdu, China). The MWM task was performed as previously described. An apparatus was a circular tank (1.2 m diameters, 50 cm in height) filled with water (23 ± 2 ℃). The tank was painted with non-toxic white paint; an escape platform (10 cm × 10 cm × 15 cm) was placed 1.5 cm below the water surface at a fixed position in a target quadrant. Different posters are plastered on the walls of the tank. A video-tracking camera above the center of the pool surface monitored the trajectory of the mice. The video signal was transmitted to a computer in an adjacent room. Before the test began, the mice were habituated to the behavior room and trained on five consecutive days, three trials per day. The mice were put into the pool from different starting points each trial of a daily training session. Each trial lasted for 60 s until the mice were out of the platform. If the mice could find the platform, it would stay on the platform for 20 s; if the mice could not find the platform in 60 s, it would be directed to the platform for 20 s. Latency time (s) to find the hidden platform was recorded after each trial of each learning session. The training lasted for 5 days; 48 h after the end of training, the hidden platform was removed. The mice were placed into the pool for the probe trial; the duration of the probe trial was 60 s; the time in the target quadrant and the crossing times were recorded.

#### Fear Conditioning Test

The experiment was performed in a white chamber (33 cm × 33 cm × 33 cm) equipped with a transparent front door, a grid floor, and a speaker. On the first day, mice were placed into the chamber for habituating for 3 min; then, a sound stimulation (20 s, 80 dB, 2000 Hz) was given followed with a short-term foot-shock (2 s, 0.8 mA) immediately. Three trials with a 60 s intertrial interval were performed in total. After 24 h, the contextual fear conditioning was evaluated. The mice were put back inside the same conditioning chamber without sound stimulation and its freezing times in 5 min were recorded. On the third day, the mice were placed into a chamber with different contextual cues, including the yellow wall, smooth plastic floor, and vinegar drops, for 5 min in total. After 2 min of free exploration, the mouse was exposed to the exact same 3-CS tones with 20 s ITI in the training stage without the foot-shock, and its freezing responses in the altered context were recorded.

#### Nissl Staining

The mice brain was sectioned into 25 μm thick slices after perfusion and fixation (4% formaldehyde). Sections were mounted on gelatin-coated slides, Nissl staining was performed according to the manufacturer’s procedure (Beyotime Biotechnology, Shanghai, China). The Nissl-stained neurons in the hippocampal CA1 or CA3 regions were counted by using the ImageJ software.

#### Golgi Staining

The mice were anesthetized by isoflurane and each mouse was perfused intracardially with 40 ml of normal saline containing 0.5% sodium nitrite. Then, the brains were removed and soaked in Golgi dye solution containing 5% potassium chromate, 5% mercuric chloride, and 5% potassium dichromate for 14 days in the dark. Next, the brains were serially sliced at 100 μm thick slices using a vibrating microtome (Leica, VT1000S, Germany). The slices were dehydrated in a gradient and transferred to a CXA solution containing formyl trichloride, xylene, and absolute ethyl alcohol (1:1:1) for 15 min. Images were observed under the microscope. Intact dendritic branches in the hippocampal DG area were selected for spine counting, and all types of spines, including mushroom, thin, and stubby spines, were counted.

### Statistical Analyses

Data were shown as the mean ± SEM and analyzed using Student’s test or one-way ANOVA by GraphPad Prism 7 software (GraphPad Software, Inc., La Jolla, CA). Student’s test (two-tailed, unpaired) was used to determine the difference among two groups. And the one-way ANOVA with Tukey’s post hoc test was used to compare with groups. *P* < 0.05 was considered statistically significant.

## Results

### DNA Damage, Chk1 Activation, and Increased CIP2A Expression in AD Human Brains and AD Mouse/Cell Models

**Fig. 1:**
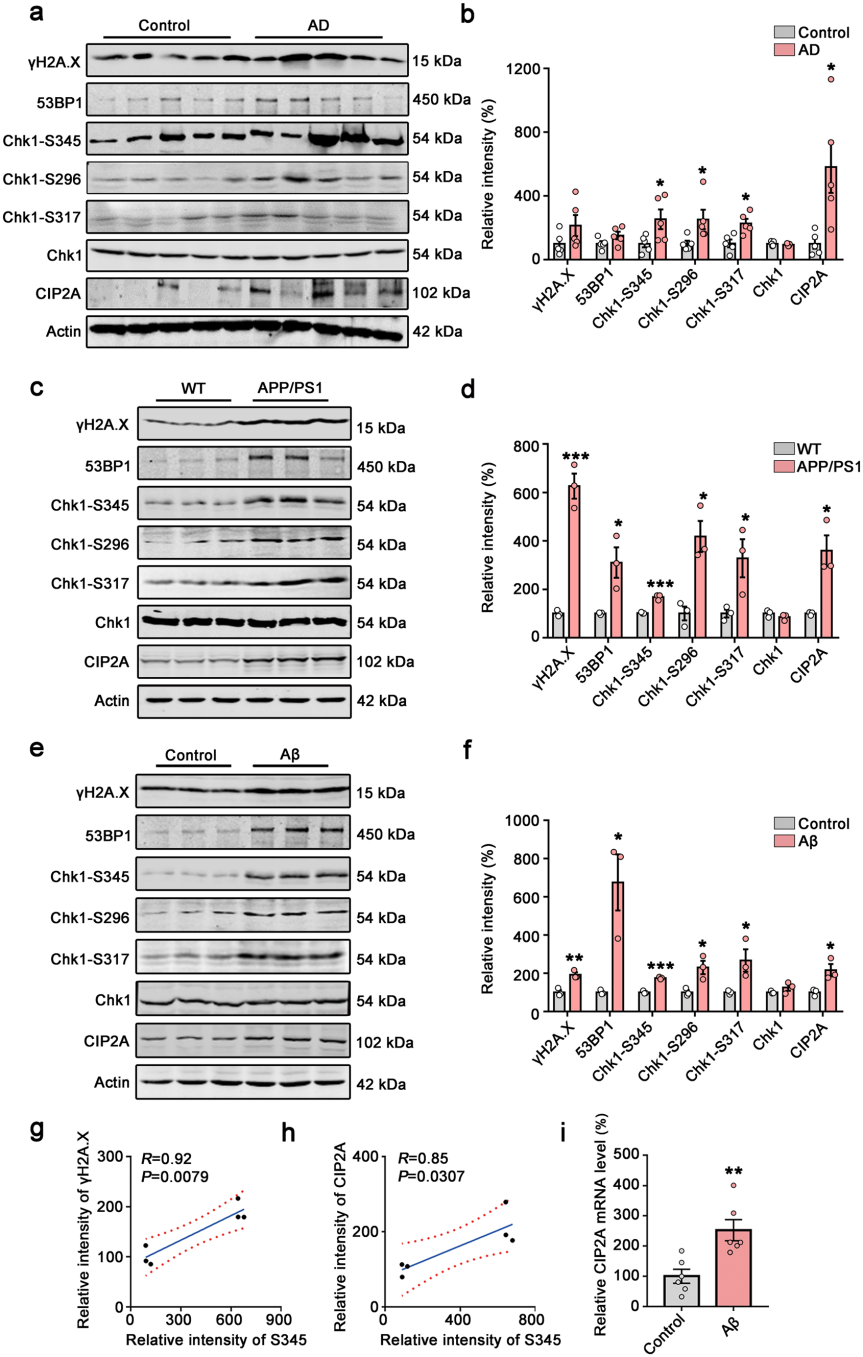
DNA damage, Chk1 activation, and increased CIP2A expression in AD human brains and AD mouse/cell models. **a** Representative immunoblots of γH2A.X, 53BP1, Chk1-S345, Chk1-S296, Chk1-S317, Chk1, CIP2A, and β-actin in AD brains. Blots were from different gels in which the same batch of samples was electrophoresed. **b** The quantitative analysis of the protein levels in **a**. Non-phosphorylated proteins such as γH2A.X, 53BP1, total Chk1, and CIP2A were normalized to the β-actin levels; phosphorylated Chk1-S345, -S296, and -S317 were normalized to total Chk1 levels. **c** Representative immunoblots of γH2A.X, 53BP1, Chk1-S345, Chk1-S296, Chk1-S317, Chk1, CIP2A, and β-actin in APP/PS1 mice brains. **d** The quantitative analysis of the protein levels in **c**. **e** Representative immunoblots of γH2A.X, 53BP1, Chk1-S345, Chk1-S296, Chk1-S317, Chk1, CIP2A, and β-actin in primary neurons with 2 µM Aβ treatment for 48 h. **f** The quantitative analysis of the protein levels in **e**. **g**–**h** Correlation analysis of Chk1-S345 and γH2A.X, Chk1-S345 and CIP2A in **e**. **i** The relative mRNA level of CIP2A in primary neurons with 2 µM Aβ treatment for 12 h. All data represent mean ± SEM, *n* = 5 in (**a**–**b**); *n* = 3 in (**c**–**i**) *P < 0.05, **P < 0.01, ***P < 0.001, compared to controls. Statistical analyses details of all data are listed in the supplementary excel sheet

To confirm the DNA damage and Chk1 activation in AD brains, we detected the protein levels of DNA damage markers (γH2A.X and 53BP1) and Chk1 and active forms of Chk1 (Chk1 phosphorylated at S345, S317, and S296), as well as CIP2A levels in brains of AD patients and APP/PS1 transgenic mice. We found that the levels of CIP2A and active Chk1 (Chk1-S345, Chk1-S317, Chk1-S296) were elevated in AD human brains and APP/PS1 transgenic mice (Fig. 1a, b, c, d). The DNA damage markers γH2A.X and 53BP1 showed a tendency of increase in AD brains (Fig. 1a, b). In APP/PS1 transgenic mice, DNA damage was significantly increased compared to control mice (Fig. 1c, d). Aβ is a major pathogenic protein in AD and induces DNA double-strand break (DSB) in primary neurons [16, 17]. We treated primary neurons with Aβ oligomer for 48 h and observed that Aβ induced DNA damage and activated Chk1 (Chk1-S345, Chk1-S317, Chk1-S296) (Fig. 1e, f). Notably, CIP2A protein levels were also increased in AD human brains and animal/cell models. Consistent with our previous results from cancer cells [5], the Chk1 phosphorylation on serine 345 was highly correlated with levels of DNA damage and CIP2A expression (Fig. 1g, h). We further detected the mRNA level of CIP2A and confirmed the increased expression of CIP2A with Chk1 activation in primary neurons treated with Aβ (Fig. 1i). Taking together, these data indicate DNA damage occurs in AD brains, and this event is accompanied with Chk1 activation and CIP2A upregulation.

### Chk1 Inhibition Reverses CIP2A Upregulation and Tau/APP Hyperphosphorylation in Aβ-Treated Primary Neurons

**Fig. 2:**
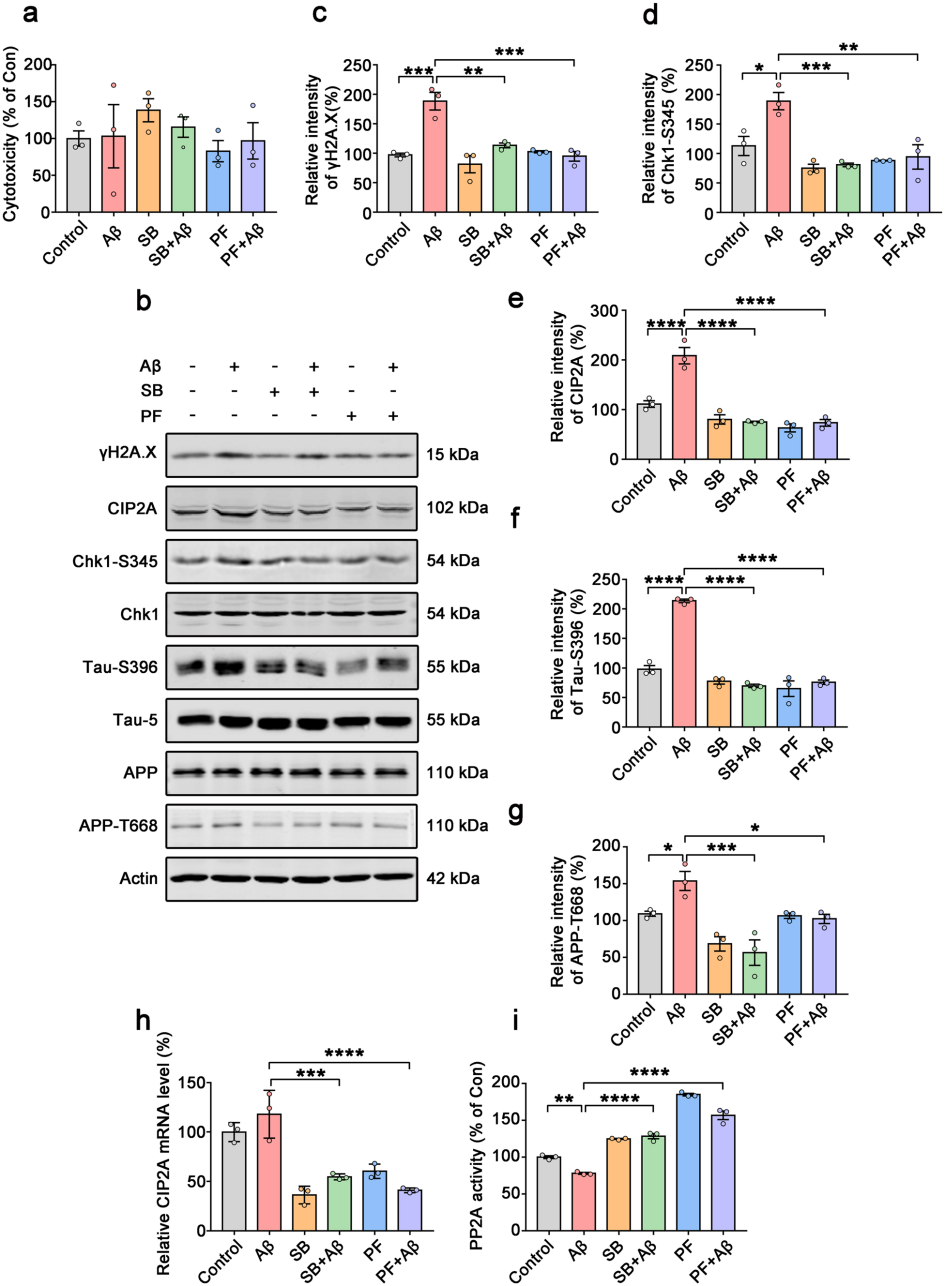
Chk1 inhibition reverses CIP2A upregulation and tau/APP hyperphosphorylation in Aβ-treated primary neurons. **a**–**i** Primary neurons were cultured with 2 µM Aβ oligomers for 48 h with or without pre-incubation of Chk1 inhibitor SB218078 (1 μM) or PF477736 (1 μM) for 48 h. **a** Cell viability was detected by LDH cytotoxicity assay kit. **b** Representative immunoblots of γH2A.X, Chk1-S345, CIP2A, Chk1, Tau-S396, Tau-5, APP, APP-T668, and β-actin in primary neurons. Blots were from different gels with the same batch of samples electrophoresed. **c**–**g** The quantitative analysis of the protein levels of γH2A.X, Chk1-S345, CIP2A, Tau-S396, and APP-T668 in **b**. Non-phosphorylated proteins such as γH2A.X and CIP2A were normalized to the β-actin levels; phosphorylated Chk1-S345, Tau-S396, and APP-T668 were normalized to corresponding total Chk1, tau (Tau-5), and APP levels respectively. **h** The relative mRNA level of CIP2A. **i** PP2A activity assay. All data represent mean ± SEM, *n* = 3, *P < 0.05, **P < 0.01, ***P < 0.001, ****P < 0.0001, comparison between the two groups

To study the role of Chk1 in mediating CIP2A upregulation in neurons, and to explore whether upregulated Chk1-CIP2A signaling induce AD-like pathogenesis, we treated primary neurons with Aβ oligomers (2 μM) for 48 h, with or without pre-incubation of Chk1 inhibitor SB218078 (1 μM) or PF477736 (1 μM) for 48 h. Even though significant DNA damage was induced upon Aβ treatment (Fig. 2b, c), there was no obvious toxicity for primary neurons either from Aβ or Chk1 inhibitors (Fig. 2a). Upon Aβ treatment–elicited DNA damage, Chk1-S345 phosphorylation was significantly increased, CIP2A expression was upregulated, and PP2A activity was inhibited. Both Chk1 inhibitors (SB218078 and PF477736) potently reversed the above changes (Fig. 2b, d, e, h, i). Notably, DNA damage was also reduced upon Chk1 inhibition (Fig. 2b, c). Tau phosphorylation (at AD-related site Ser396) and APP phosphorylation (at Thr668) were increased by Aβ oligomers incubation, which were also reversed by Chk1 inhibitors (Fig. 2f, g). These results indicate that Chk1 activation mediates the DNA damage–induced CIP2A overexpression, PP2A inhibition, and tau/APP hyperphosphorylation. APP phosphorylation at Thr668 increases its interaction with BACE1 and in turn promotes Aβ production [18–20]; thus, Chk1 activation may play a key role in this disease-promoting positive feedback loop.

We further used another cell model to confirm this hypothesis. Oxidative stress can induce DNA damage. We treated primary neurons with hydrogen peroxide for 2 h to simulate cellular DNA damage response, with or without pre-incubation of Chk1 inhibitor SB218078 (1 μM) or PF477736 (1 μM) for 48 h. Similar with the previous results, hydrogen peroxide induced Chk1 activation, CIP2A overexpression, suppression of PP2A activity, and tau hyperphosphorylation, while Chk1 inhibition reversed these changes. Again, in this cell model, the Chk1 activation (Chk1-S345) was highly correlated with increased DNA damage and increased CIP2A expression (Fig. S1). Thus, at the cellular level, DNA damage promotes AD-like pathology via the Chk1-CIP2A-PP2A signaling axis.

### Chk1 Overexpression Induces Cognitive Deficits in Mice

**Fig. 3:**
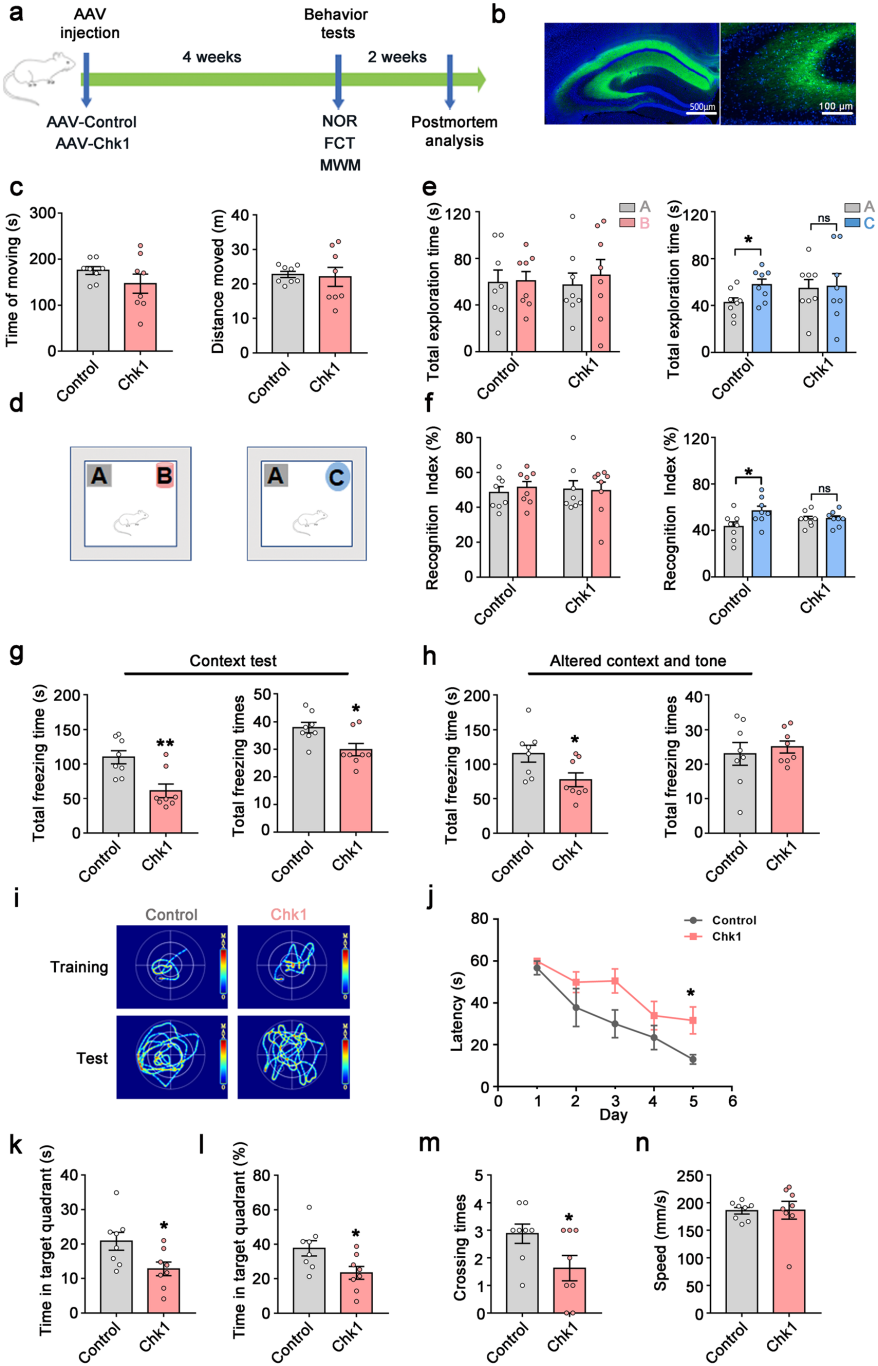
Chk1 overexpression induces cognitive deficits in mice. **a**–**n** Sixteen mice aged 8 weeks were divided into two groups and injected with AAV-Con and AAV-Chk1virus respectively into the bilateral ventricles. After 4 weeks, all mice were tested by different behavioral test paradigms. **a** A schematic diagram for the behavioral tests (novel objection recognition test (NOR), fear conditioning test (FCT), Morris water maze test (MWM)) of the mice. **b** The infection of the virus in the hippocampus was confirmed by fluorescence microscopy. **c** The total time (s) and distance (m) of moving in an open field test. **d** The experimental design of novel object recognition test (NOR). Left is the acquisition trial. The test trial was conducted 24 h (right) after the acquisition trial. **e** Left: the total exploration time (s) to object A and object B in the acquisition trial; right: the total exploration time (s) to object A and object C in the test trial. **f** Left: the recognition index to object A and object B in the acquisition trial; right: the recognition index to object A and object C in the test trial. **g** The total freezing time (s) and freezing times of the mice in the context test. **h** The total freezing time (s) and freezing times of the mice in the altered context and tone test. **i** The representative searching trace on day 5 of the training and the probe trial at 48 h after training in MWM. **j** The latency of the mice to find the hidden platform. **k**–**n** The time in the target quadrant (s) (**k**), the percentage of time in the target quadrant (**l**), the crossing times (**m**), the swimming speed (**n**) of the mice. All data represent mean ± SEM, *n* = 8, *P < 0.05, **P < 0.01, compared to controls

**Fig. 4:**
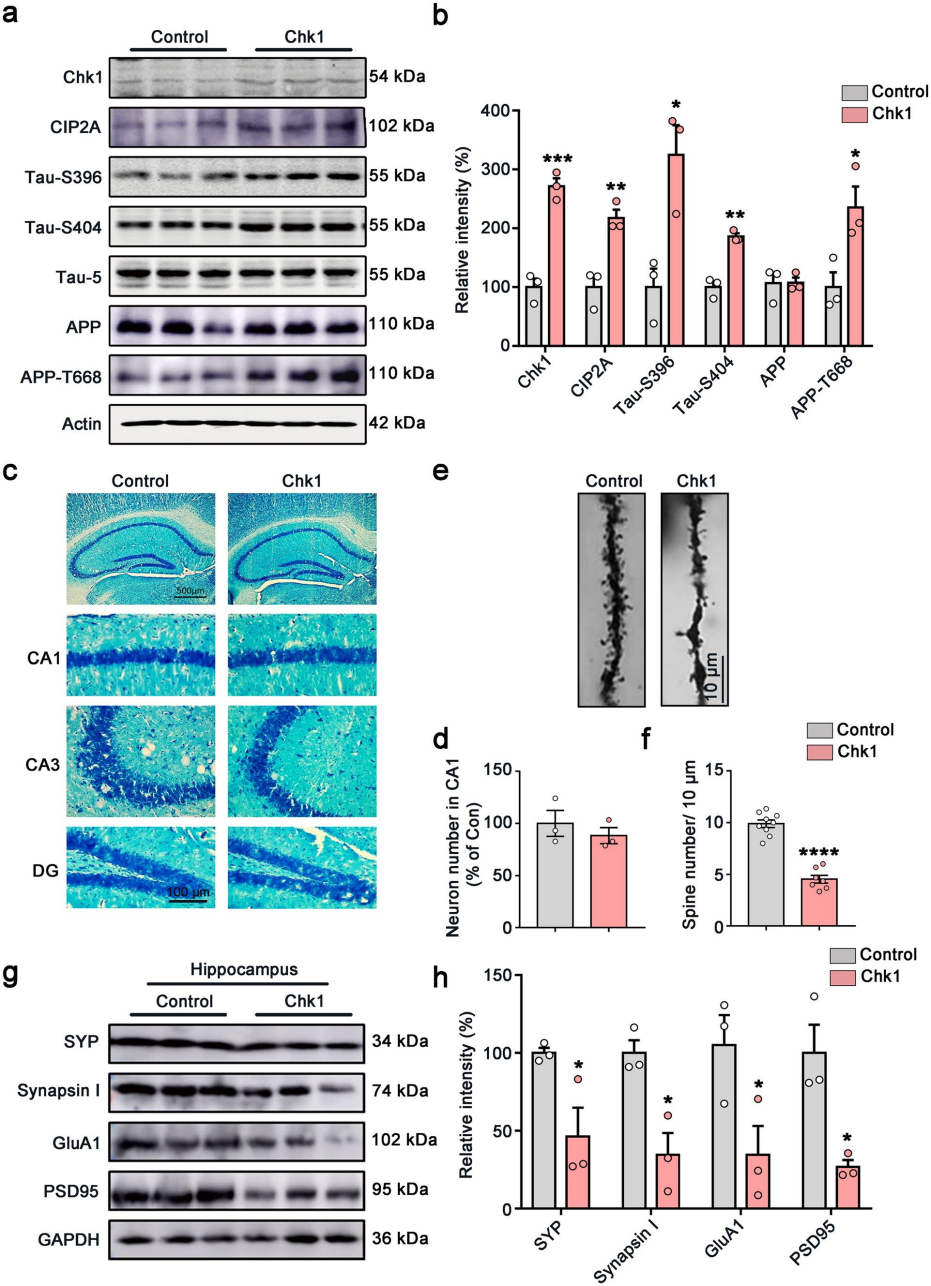
Overexpression of Chk1 in neurons induces CIP2A upregulation, tau/APP hyperphosphorylation, and synaptic impairments in mice. **a** Representative immunoblots of Chk1, CIP2A, Tau-S396, Tau-S404, Tau-5, APP, APP-T668, and β-actin in the hippocampus of the mice. Blots were from different gels with the same batch of samples electrophoresed. **b** The quantitative analysis of the protein level in **a**. Non-phosphorylated proteins such as Chk1, CIP2A, and APP were normalized to the β-actin levels; phosphorylated Tau-S396, Tau-S404, and APP-T668 were normalized to corresponding total tau (Tau-5) and APP levels respectively. **c** Representative images of Nissl staining of the mice brain slices. **d** The quantitative analysis of the neuron number in the CA1 region of **c**. The cell number was counted in brain slices from three mice in each group; one brain slice was counted for each mouse, and the neuron number in the CA1 region was counted by using the ImageJ software. **e** Representative picture in Golgi staining of the mice. **f** The quantitative analysis of the spine number in **e** from 3 brains. Spines in 9–10 intact dendrites in the hippocampal DG area were counted in each group. **g** Representative immunoblots of synaptic proteins (SYP, Synapsin I, GluA1 and PSD95) and GAPDH in the hippocampus of the mice. Blots were from different gels with the same batch of samples electrophoresed. **h** The quantitative analysis of the protein level in **g**. Bands intensity was normalized to GAPDH. All data represent mean ± SEM, *n* = 3, *P < 0.05, **P < 0.01, ***P < 0.001, ****P < 0.0001, compared to controls

Next, to verify the role of neuronal Chk1 activation in AD pathogenesis, we explored the effect of Chk1 overexpression in vivo. We injected the AAV-Con or AAV-Chk1 virus into the bilateral ventricles of C57/BL6 mice aged 8 weeks (Fig. 3a). Both viruses carry the Syn promoter to ensure the expression of Chk1 in mature neurons. After 4 weeks, overexpression of Chk1 in the hippocampus was confirmed by fluorescence microscopy (Fig. 3b). Progressive cognitive and memory impairments are the main clinical features of AD [21]. To evaluate the effect of Chk1 overexpression on the learning and memory of mice, several behavioral tests were performed. In an open field test, there was no significant difference in the time and distance of moving between two groups, indicating overexpression of Chk1 has no effect on the motor ability of mice (Fig. 3c). In the novel objection recognition test (NOR) (Fig. 3d), the total exploration time and the recognition index were comparable between the two groups in the acquisition trial. During the test trial, 24 h after the acquisition trial, mice overexpressing of Chk1 didn't show increased interest to the new object, and their total exploration time and recognition index to the new object were significantly lower than that of control mice, which indicated impaired cognitive function in the Chk1 overexpressing mice (Fig. 3e, f). We further performed a fear conditioning test to assess the fear memory. The result showed that the mice overexpressing of Chk1 had decreased total freezing time (s) and total freezing times in the context paradigm compared with the control mice (Fig. 3g). In the tone conditioning paradigm, the Chk1 mice also showed a significant reduction in freezing time (s) (Fig. 3h). Next, we explored the impact of Chk1 activation on spatial learning and memory ability by the Morris water maze test (MWM). In the MWM test, the mice were trained for 5 days with three trials per day, we found that the Chk1 mice showed impaired learning ability on day 5 with significantly longer latency to find the hidden platform (Fig. 4i, j). In memory testing on day 7, the mice overexpressing Chk1 spent less time in the target quadrant and showed decreased crossing times over the platform location compared with control mice (Fig. 3k, l, m). The motor ability showed no significant difference between the two groups reflected by swimming speed (Fig. 3n). Taken together, these results indicate that activation of Chk1induces cognitive and memory deficits in mice.

### Chk1 Overexpression in Neurons Induces CIP2A Upregulation, Tau/APP Hyperphosphorylation, and Synaptic Impairments in Mice

To validate whether the Chk1-CIP2A-PP2A signaling axis was induced by neuronal Chk1 overexpression in vivo, we detected the changes of these proteins in the mice brains aged 12–14 weeks. Consistently with the cell culture results, overexpression of Chk1 in mouse brains induced tau hyperphosphorylation (at Ser396 and Ser404) and APP-T668 hyperphosphorylation accompanied with increased expression of CIP2A (Fig. 4a, b).

Synaptic impairment is an early event in the process of AD [22–24], which underlies cognitive dysfunction. Both Aβ oligomer and pathological hyperphosphorylated tau have synaptic toxicity. We have previously shown that neuronal CIP2A overexpression results in synaptic impairment and dysfunction [6]. Thus, we further detect whether synaptic damage is induced by Chk1 activation in the present study. Nissl staining showed that Chk1 overexpression did not affect the number of neurons in the hippocampus CA1 region (Fig. 4c, d). However, Chk1 overexpression caused a marked decrease in spine density compared with control mice suggested by Golgi staining (Fig. 4e, f). Western blotting results indicated that the level of pre-synaptic proteins including synapsin I and synaptophysin (SYP), as well as postsynaptic proteins including GluA1 and PSD95, was significantly reduced in the Chk1 group (Fig. 4g, h), further confirming the synaptic impairment. In summary, these data validate that Chk1 overexpression causes CIP2A induction and AD-like pathological features, including synaptic impairment and tau/APP hyperphosphorylation in vivo.

### Chk1 Inhibitor (GDC-0575) Reduces CIP2A Expression, Activates PP2A, and Decreases Tau Phosphorylation and Aβ Levels in AD Cell Models

**Fig. 5:**
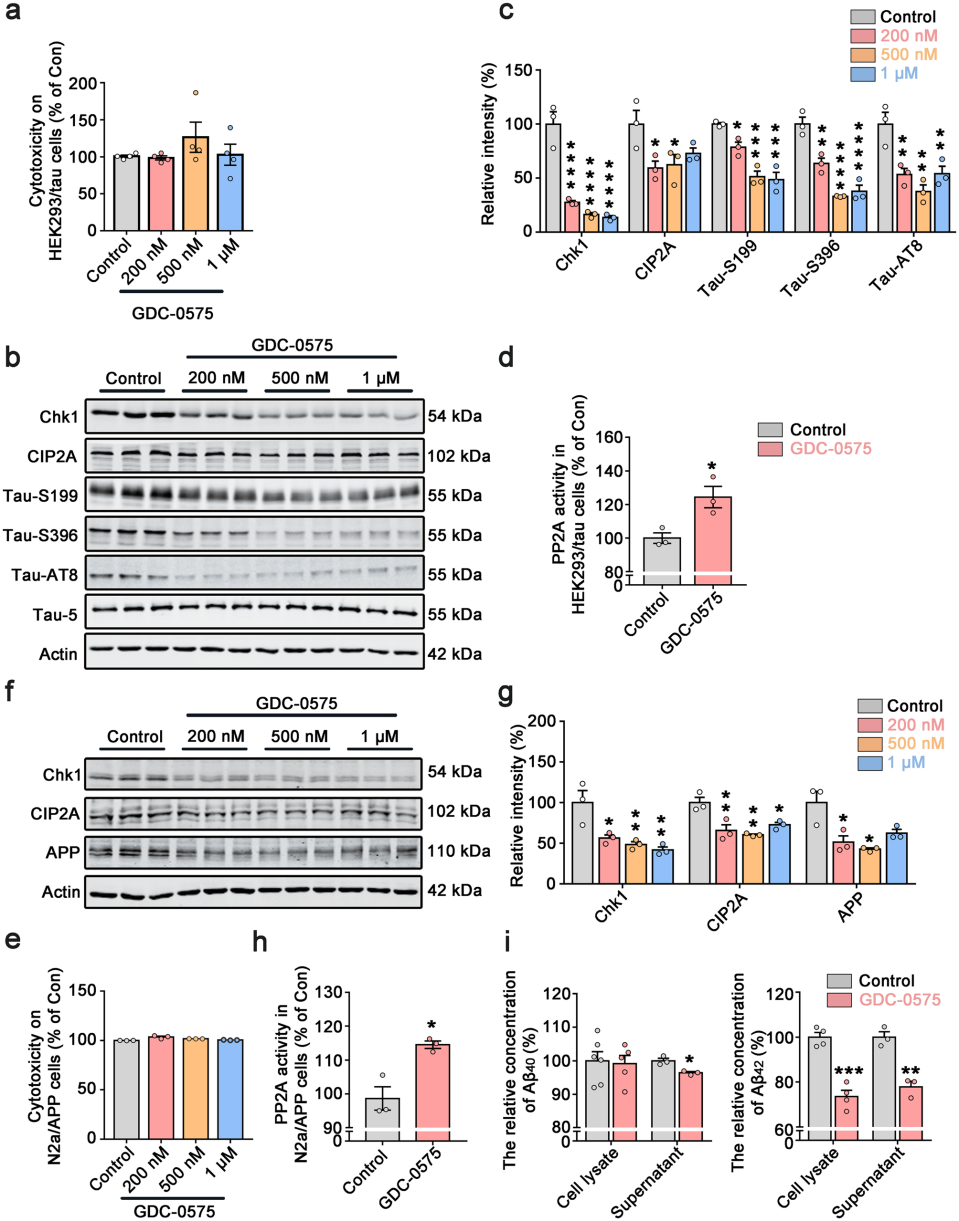
Chk1 inhibitor (GDC-0575) reduces CIP2A expression, activates PP2A, decreases tau phosphorylation and Aβ levels in AD cell models. **a** Cell viability was detected in HEK293/tau cells by LDH cytotoxicity assay kit. **b** Representative immunoblots of Chk1, CIP2A, Tau-S199, Tau-S396, Tau-AT8, Tau-5, and β-actin in HEK293/tau cells treated with increasing doses of GDC-0575 (0, 0.2, 0.5, 1 μM) for 24 h. Blots were from different gels with the same batch of samples electrophoresed. **c** The quantitative analysis of the protein levels in **b**. Non-phosphorylated proteins such as Chk1, CIP2A, and APP were normalized to the β-actin levels; phosphorylated Tau-S199, -S396, and -AT8 were normalized to total tau (Tau-5) levels. **d** PP2A activity was detected in HEK293/tau cells. **e** Cell viability was detected in N2a/APP cells by LDH cytotoxicity assay kit. **f** Representative immunoblots of Chk1, CIP2A, APP, and β-actin in N2a/APP cells treated with increasing doses of GDC-0575 (0, 0.2, 0.5, 1 μM) for 24 h. **g** The quantitative analysis of the protein levels in **f**. **h** PP2A activity was detected in N2a/APP cells. **i** The relative concentration of Aβ_40_ in cell lysate and supernatant (left) and Aβ_42_ in cell lysate and supernatant (right) of N2a/APP cells treated with 0.5 µM GDC-0575 treatment for 24 h. All data represent mean ± SEM, **a**–**h** *n* = 3, **i** the relative concentration of Aβ_40_ and Aβ_42_ in the cell lysate of the control group, *n* = 6, *n* = 4 respectively, and in the GDC group, *n* = 5, *n* = 4 respectively. **i** The relative concentration of Aβ_40_ and Aβ_42_ in supernatant of control group and GDC-0575 group *n* = 3, *P < 0.05, **P < 0.01, ***P < 0.001, ****P < 0.0001, compared to controls

Our findings that Chk1 activation promotes tau/APP hyperphosphorylation and cognitive dysfunction indicate that pharmacological Chk1 inhibition could reverse AD-like pathological features in DNA-damaged neurons and in the animal model. To test the potential therapeutic impact of Chk1 inhibition in AD, we used less toxic and more potent second-generation Chk1 inhibitor GDC-0575 [25, 26] (MCE, HY-112167) to treat cultured HEK293/tau cells and N2a/APP cells. Cells were incubated with GDC-0575 at different concentrations for 24 h. LDH assay results suggested that the Chk1 inhibitor did not cause significant cellular toxicity (Fig. 5a, e) at used concentrations. In both cell lines, GDC-0575 reduced Chk1 protein levels dose-dependently. With Chk1 inhibition, GDC-0575 at all concentrations used significantly reduced CIP2A levels and decreased tau phosphorylation (at Ser199, Ser396, and AT8 sites) and APP levels (Fig. 5b, c, f, g). We further confirmed that GDC-0575 induced a significant upregulation of PP2A activity (Fig. 5d, h). At last, GDC-0575 reduced Aβ_42_ levels in N2a/APP cells (Fig. 5i). These results suggest that Chk1 inhibitor GDC-0575 effectively inhibits the Chk1-CIP2A-PP2A signaling axis and prevents AD pathologic changes in cell models.

### Chk1 Inhibitor (GDC-0575) Ameliorates Cognitive Deficits in APP/PS1 Mice

**Fig. 6:**
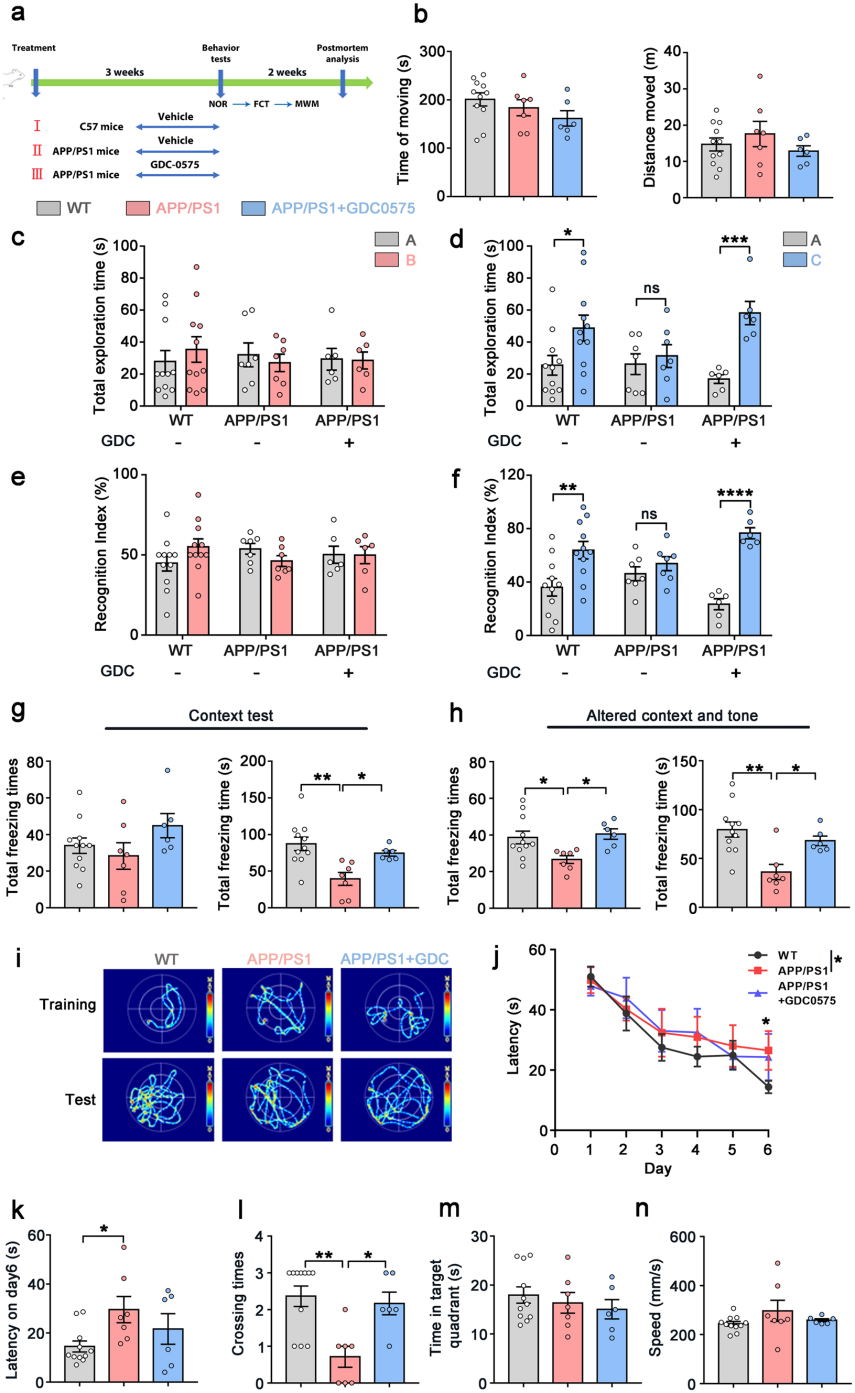
Chk1 inhibitor (GDC-0575) ameliorates cognitive deficits in APP/PS1 mice. **a** A schematic diagram for the animal treatment and behavioral tests (novel objection recognition test (NOR), fear conditioning test (FCT), Morris water maze test (MWM)). **b** The total time (s) and distance (m) of moving in an open field test. **c** The total exploration time to object A and object B in the acquisition trial. **d** The total exploration time to object A and object C in the test trial at 24 h after training. **e** The recognition index to object A and object B in the acquisition trial. **f** The recognition index to object A and object C in the test trial at 24 h after training. **g** The total freezing time (s) and freezing times of the mice in the context test. **h** The total freezing time (s) and freezing times of the mice in the altered context and tone test. **i** Representative searching trace on day 6 of the training and the probe trial at 48 h after training. **j**–**k** The latency of the mice to find the hidden platform. **l**–**n** The crossing times (**l**), the time in the target quadrant (s) (**m**), the swimming speed (**n**) of the mice. All data represent mean ± SEM, WT (treated with saline solution) *n* = 11, APP/PS1 (treated with saline solution) *n* = 7, APP/PS1 (treated with GDC-0575) *n* = 6, *P < 0.05, **P < 0.01, ***P < 0.001, ****P < 0.0001, comparison between the two groups

Based on the observed potent effects of GDC-0575 on ameliorating Alzheimer-like pathologic changes in cells, we further explored whether this drug can prevent AD progress in animal models. In animal experiments, age-matched wild-type mice (WT) and APP/PS1 mice were treated with intragastric administration of saline solution or GDC-0575 at the concentration of 25 mg/kg twice a week, which lasted for 3 weeks. The dosage of GDC-0575 was determined according to the concentrations used in clinical trials of solid tumor therapy and other animal experiments [25, 27] (Fig. 6a). Open field test results showed that there was no difference in the time and distance of moving among the three groups (Fig. 6b). In the novel objection recognition test (NOR), the total exploration time and the recognition index of the three groups showed no difference in the acquisition trial (Fig. 6c, e). In the test trial, the total exploration time and the recognition index to the new object were significantly decreased in APP/PS1 mice compared with WT mice, while GDC-0575 treatment restored the cognition to the new object in APP/PS1 mice (Fig. 6d, f). We further executed a fear conditioning test to assess the contextual fear memory and found that the total freezing time (s) of APP/PS1 mice was less than the WT mice and the GDC-0575 reversed the loss of fear memory in APP/PS1 mice both in the context paradigm and the tone conditioning paradigm (Fig. 6g, h). To explore the role of GDC-0575 in restoring spatial learning and memory ability in APP/PS1 mice, we performed the Morris water maze test. The results revealed that there was no significant difference in swimming speed among the three groups and APP/PS1 mice had longer latency than WT mice on the training day 6 (Fig. 6i, j, k, n). In the test trial, APP/PS1 mice showed memory impairment compared with the WT group suggested by decreased crossing times over the platform. The spatial learning and memory deficits in APP/PS1 mice were rescued by GDC-0575 treatment (Fig. 6l). The time in the target quadrant had no difference among the three groups (Fig. 6m). In general, the data support that Chk1 inhibitor (GDC-0575) ameliorates cognitive deficits in APP/PS1 mice.

### Chk1 Inhibitor (GDC-0575) Rescues Neuron Loss and Synaptic Impairments in APP/PS1 Mice

**Fig. 7:**
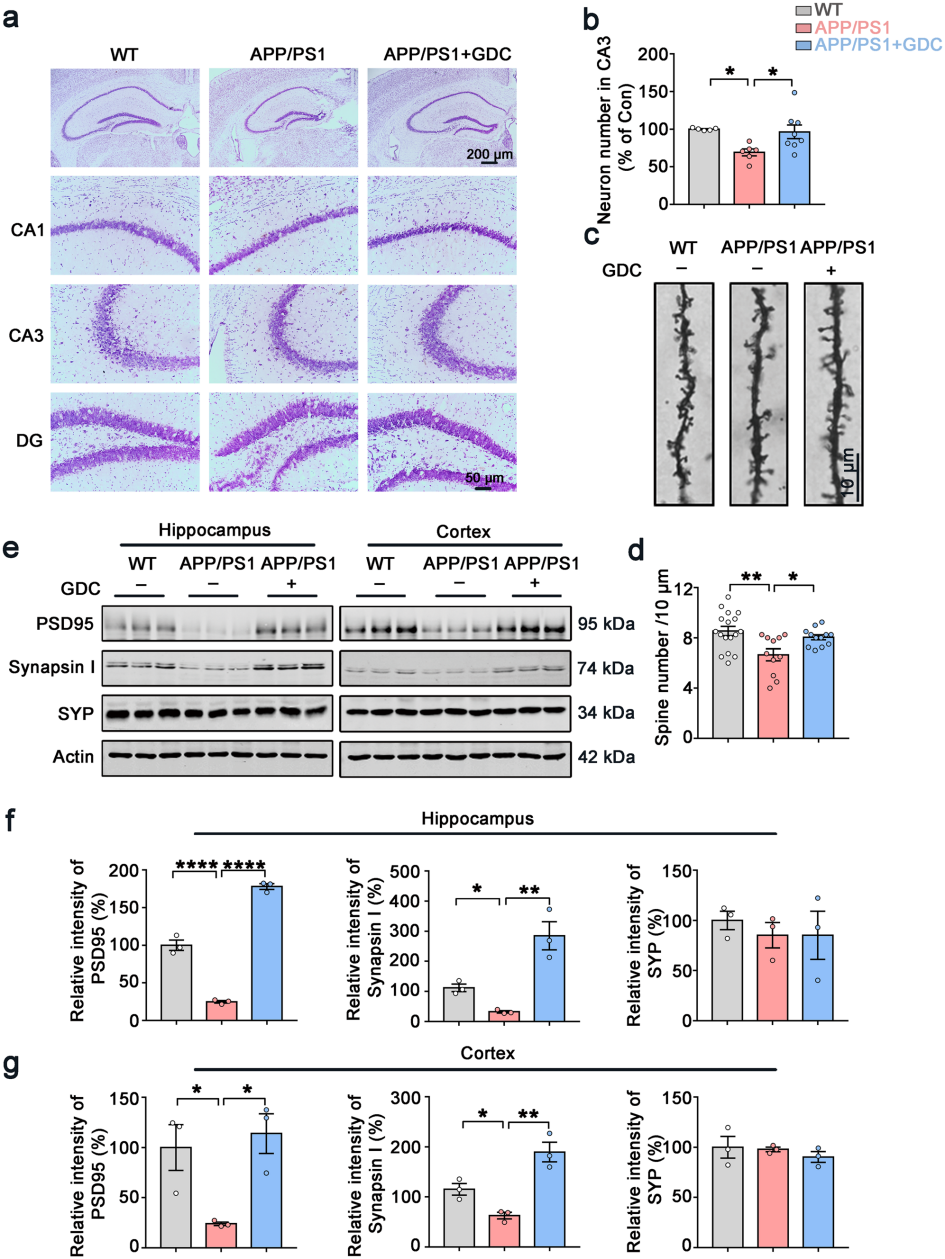
Chk1 inhibitor (GDC-0575) rescues neuron loss and synaptic impairments in APP/PS1 mice. **a** Representative images of Nissl staining of the mice brain slices. **b** The quantitative analysis of the neuron number in the CA3 region of **a**. The cell number in the hippocampal CA3 area was counted in 2–3 brain slices from each mouse (*N* = 3) in each group. **c** Representative images of Golgi staining of the mice hippocampal slices. **d** The quantitative analysis of the spine number in **c**. Spines in 11–17 intact dendrites in the hippocampal DG area were counted in each group. **e** Representative immunoblots of synaptic proteins (PSD95, Synapsin I, Synaptophysin (SYP), and β-actin) in the hippocampus and cortex of the mice. Blots were from different gels with the same batch of samples electrophoresed. **f** The quantitative analysis of the protein levels in the hippocampus in **e**. Bands intensity was normalized to β-actin levels. **g** The quantitative analysis of the protein levels in brain cortex in **e**. All data represent mean ± SEM, *n* = 3, *P < 0.05, **P < 0.01, ***P < 0.001, ****P < 0.0001

Next, we evaluated the neuronal and synaptic impairments in the three groups of mice. The Nissl staining result showed GDC-0575 treatment could reverse the neuron loss in hippocampal CA3 region of APP/PS1 mice (Fig. 7a, b). Golgi staining suggested that the density of dendritic spines was significantly reduced in APP/PS1 mice compared with control mice. GDC-0575 treatment partially reversed the loss of dendritic spines in APP/PS1 mice (Fig. 7c, d). Consistent with these findings, the levels of pre-synaptic protein Synapsin I and postsynaptic protein PSD95 in the hippocampus and cortex of APP/PS1 mice with GDC-0575 treatment were significantly increased (Fig. 7e, f, g). Pre-synaptic protein synaptophysin (SYN) showed no difference among the three groups, indicating that SYN is less vulnerable to damage, or is more able to be compensatory-upregulated during disease progression. Similar results could be observed in other pieces of research [28, 29]. Taken together, our results showed that Chk1 inhibitor (GDC-0575) rescued neuron loss and synaptic impairments in APP/PS1 mice.

### Chk1 Inhibitor (GDC-0575) Ameliorates CIP2A Upregulation, Tau Hyperphosphorylation, and Aβ Overproduction in APP/PS1 Mice

**Fig. 8:**
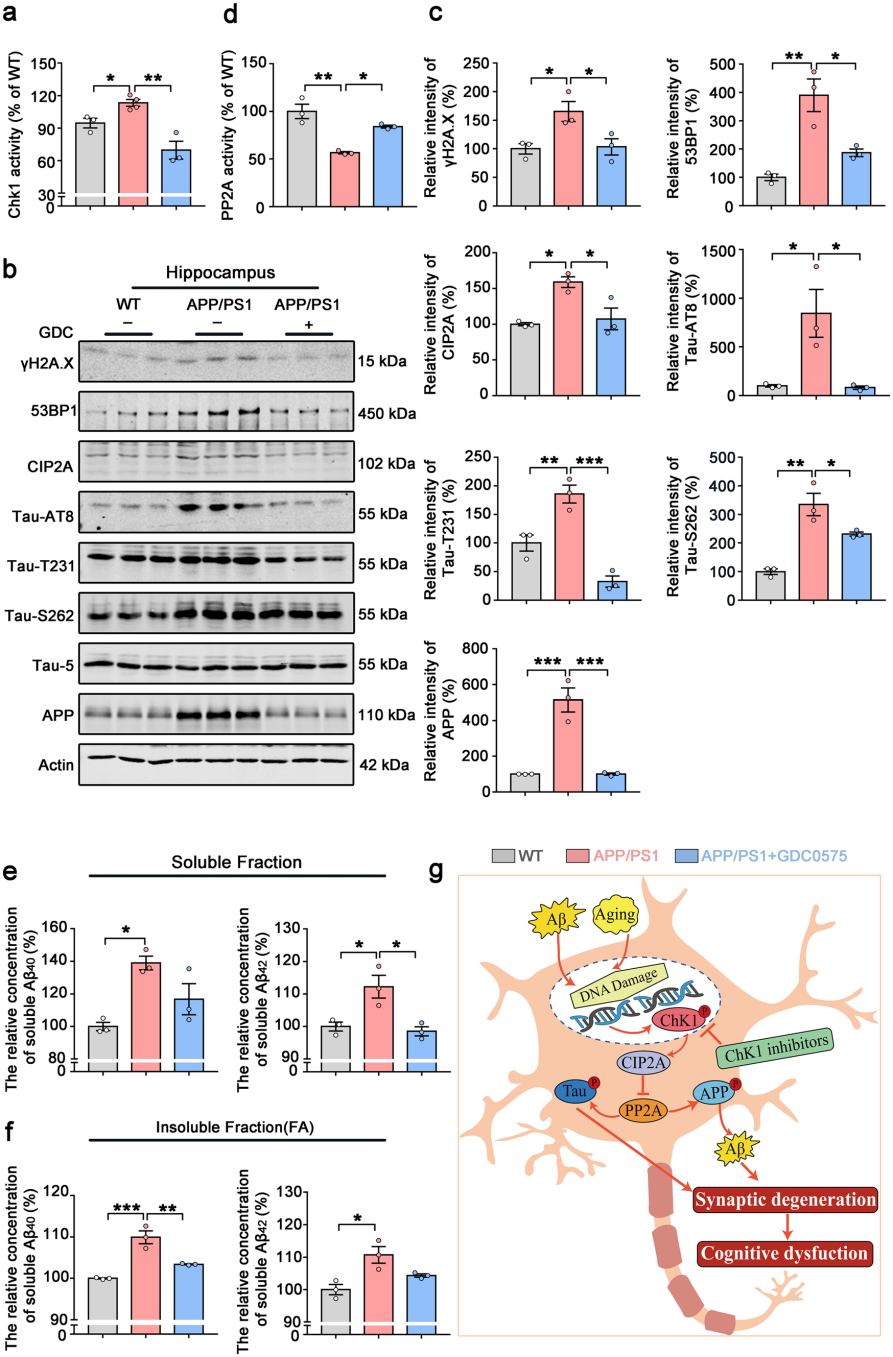
Chk1 inhibitor (GDC-0575) ameliorates CIP2A upregulation, tau hyperphosphorylation and Aβ production in the hippocampus of APP/PS1 mice. **a** Chk1 activity was detected in the hippocampus of the mice. **b** Representative immunoblots of γH2A.X, 53BP1, CIP2A, Tau-AT8, Tau-T231, Tau-S262, Tau-5, APP, and β-actin in the hippocampus of the mice. Blots were from different gels with the same batch of samples electrophoresed. **c** The quantitative analysis of the protein level in **b**. Non-phosphorylated proteins such as γH2A.X, 53BP1, CIP2A, and APP were normalized to the β-actin levels; phosphorylated Tau-S262, Tau-AT8, and Tau-T231 were normalized to total tau (Tau-5) levels. **d** PP2A activity was detected in the hippocampus of the mice. **e** The soluble Aβ_40_ and Aβ_42_ in the mice hippocampal tissues were detected by ELISA kit. **f** The insoluble Aβ_40_ and Aβ_42_ in the mice hippocampal tissues were detected by ELISA kit. All data represent mean ± SEM, **a** APP/PS1 group *n* = 4, **b**–**f** *n* = 3, *P < 0.05, **P < 0.01, ***P < 0.001, comparison between the two groups. **g** Summary of the role of Chk1 activation in promoting AD-like pathologies through CIP2A/PP2A signaling

At last, to further confirm that the therapeutic effect of Chk1 inhibitor GDC-0575 in the AD mice is through targeting the Chk1-CIP2A-PP2A-tau/APP signaling axis, we detected the changes of these key proteins in the mouse models. We first confirmed that Chk1 was activated in APP/PS1 mice and this was inhibited by GDC-0575 (Fig. 8a). Consistent with the changes of Chk1 activities, the expression of CIP2A was increased, and DNA damage and PP2A activity were depressed in APP/PS1 mice, both of which were rescued by GDC-0575 treatment (Fig. 8b, c, d). The increased APP and tau phosphorylation at AT8, Tau-T231, and Tau-S262 sites in AD mice were also reversed by Chk1 inhibition (Fig. 8b, c). Similar changes were detected in the brain cortex (Fig. S2). Aβ levels were significantly increased both in the soluble and insoluble fractions of APP/PS1 mice brain tissues, which was also reversed by GDC-0575 administration (Fig. 8e, f). Collectively, these results validate our in vitro findings that pharmacological Chk1 inhibition results in inhibition of CIP2A expression, reactivation of PP2A phosphatase activity, leading to the reverse of the tau hyperphosphorylation and Aβ overproduction (Fig. 8g).

## Discussion

Alzheimer’s disease is a devastating neurodegenerative disease with limited treatment strategies at current. The development of effective therapy depends on the discovery of key disease mechanisms. The neuropathology of AD is characterized by the intraneuronal neurofibrillary tangles (NFTs) formed by aggregated hyperphosphorylated microtubule-associated protein tau and the extracellular accumulation of amyloid-β (Aβ) peptide into amyloid plaques [2, 30, 31]. The accumulation and aggregation of these two proteins result in neurotoxicity and neuroinflammation, which play a central role in the disease progression [32]. In the last decade, great efforts were dedicated to the development of drugs targeting tau or Aβ [33–37]. In the present study, we disclose a new signaling pathway participating in both tauopathy and Aβ overproduction in Alzheimer’s disease. Based on this finding, a new potential drug target Chk1 in AD, which is also an “old” but important drug target in cancer, was unearthed.

Chk1 is a DNA damage response kinase which is phosphorylated and activated upon DNA damage [38, 39]. Both DNA double-strand break (DSB) and single-strand break (SSB) can induce Chk1 activation through phosphorylation by different DNA damage sensor kinases such as ATR, ATM, and DNA-PK in different DNA repair pathways [40]. In proliferating cells, activation of Chk1 causes phosphorylation of several important cell cycle regulatory proteins such as cdc25A, cdc25C, and p53, resulting in cell cycle arrest. Chk1 also participates in DNA repair through phosphorylating and regulating key DNA repair components such as p53, BRCA2, and RAD51 [7, 10]. In lots of human tumors, Chk1 is abnormally activated [41–45], and dozens of Chk1 inhibitors were designed and developed for cancer treatment [7, 46]. Most of these drugs are still in developing and some of them are currently in clinical trials. The therapeutic effect of Chk1 inhibitors in tumor is believed to be through compromising replication stress (RS)-triggered ATR-Chk1 signaling on which cancer cells are highly dependent [47–51]. ATR-Chk1 signal also promotes homologous recombination repair (HRR). Therefore, inhibiting Chk1 has the potential to compromise HRR, thereby sensitizing cells to DNA damaging anticancer agents. Both therapeutic effects highly relate to DNA replication which is a common event in cancer cells.

Both neurons and glia are suffering from accumulating DNA damage in AD brains. Different forms of DNA damage, such as SSB and DSB, were detected [11]. Through immunostaining of DSB marker γH2AX in the brains of MCI or AD and cognitively unimpaired controls, Shanbhag et al. [13] observed an accumulation of DSBs in vulnerable neuronal and glial cell populations in AD from early stages onward. In addition, physical neuronal activity induces transient DSBs, and Aβ exacerbates DNA damage. In Aβ-overloaded hAPP mice, DSB levels are significantly increased [17]. Based on these facts, it will be interesting to explore the role of DNA damage in mediating AD pathologies.

As an important DDR kinase, is Chk1 activated in response to DNA damage in AD brains? If it is, what is the role of Chk1 activation in AD development? With this question in mind, we first detected the Chk1 activities in AD human brains and animal/cell models. Chk1 is phosphorylated on both serines 317 and 345 via ATM/ATR-mediated mechanisms [52, 53]. Alternatively, another DNA damage sensor DNA-PK also phosphorylates Chk1 on serine 345 and activates it [8]. Serine 296 is an autophosphorylation site to help the kinase achieve full activation. We detected the phosphorylation levels of Chk1 on these three active sites and found that with DNA damage, Chk1 was activated in AD human brains, APP/PS1 mouse hippocampus, and Aβ-treated primary neurons. Notably, DNA damage (γH2A.X level) highly correlated with Chk1 activation (Ser 345 phosphorylation level), indicating a causal relationship. To further identify that DNA damage induces Chk1 activation in neurons, we treated cultured neurons with H_2_O_2_ to increase DNA damage and also observed consistent Chk1 activation. These results indicate that DNA damage induces Chk1 activation in AD neurons.

CIP2A is an endogenous inhibitor of PP2A. In our previous study, we have identified that CIP2A is upregulated in AD brains and promotes AD-like tauopathy and amyloidosis through inhibiting PP2A’s dephosphorylating effects on tau/APP [5]. Notably, in tumor cells, Chk1 activation is upstream of CIP2A overexpression in response to chronic DNA damage [8]. Thus, it is possible that in AD brains, activated Chk1 is a key factor that links DNA damage and CIP2A overexpression. To identify this hypothesis, we used Chk1 inhibitors to prevent Chk1 activation in Aβ-treated primary neurons, and overexpressed Chk1 in mouse brains. We found that Chk1 inhibition efficiently reversed CIP2A overexpression, PP2A inhibition, and tau/APP hyperphosphorylation. Similar results were observed in H_2_O_2_-treated neurons with pre-incubation of Chk1 inhibitors. On the contrary, Chk1 overexpression in mouse brains resulted in CIP2A upregulation and tau/APP hyperphosphorylation. These results suggest that DNA damage–induced Chk1 activation induces downstream CIP2A-PP2A-tau/APP phosphorylation signaling. Hyperphosphorylated tau and Aβ are toxic to synapses and neurons. Consistent with our previous findings in CIP2A-overexpressed mice, the Chk1 overexpression resulted in remarkable synaptic dysfunction and severe cognitive impairment in mice, which mimics the changes in AD patients.

Based on the finding that Chk1 activation enhances CIP2A-PP2A-tau/APP phosphorylation signaling in AD brains, we propose Chk1 as a novel druggable target for AD treatment. Though in tumor cells, Chk1 inhibition significantly promotes cell death, the possibility for occurrence of a similar event in neurons is very low, since DNA replication and replication stress are absent in neurons. A typical example is that medulloblastoma cells showed hypersensitivity to pharmacological Chk1 inhibition, which likely results from enhanced damage to intracellular organelles, elevated replicative stress and DNA damage, and activation of apoptosis. While in the same research, Chk1 inhibition only caused minor toxicity in primary rat neurons in vitro [54]. Another two studies reported that cultured embryonic cortical neurons had a significant basal activity of Chk1, inhibition of the normal physiological activity of Chk1 by inhibitor UCN-01 or DNA damaging agent camptothecin results in decreased cell viability [55, 56]. These data indicate that the normal Chk1 activity is required for the survival of neurons. However, in AD brains, Chk1 is abnormally activated. Just like the numerous other kinase targets in disease treatment, proper inhibition of the kinase activity can achieve the aim of preventing the disease progression without impairing the normal function of cells.

Thus, we used the second-generation Chk1 inhibitor GDC-0575 for the exploration of targeting Chk1 in AD cell and animal models. The results showed that GDC-0575 at all concentrations used (200, 500, 1000 nM) has no toxicity to cultured HEK293/tau and N2a/APP cells. While at a concentration as low as 200 nM, GDC-0575 potently reduced CIP2A expression, decreased tau phosphorylation and APP levels. Further investigation confirmed the activation of PP2A and reduction of Aβ production in GDC-0575 treated cells. In APP/PS1 mice, abnormal Chk1 activation was identified by direct Chk1 activity assay. Oral gavage of GDC-0575 resulted in significant inhibition of Chk1 in the mouse brain, indicating that GDC-0575 could have a pharmacological effect on CNS. With inhibition of Chk1 in AD mice, cognitive function was ameliorated, and the upregulated CIP2A-PP2A-tau/APP signaling axis was reversed. Correspondingly, neuron loss and synaptic impairments were rescued. Notably, Chk1 inhibitors used in the present study including SB218078 and PF477736 could reduce the DNA damage in neurons instead of increasing DNA damage in tumor cells, which was manifested by decreased γH2A.X level, indicating different therapeutic mechanisms of Chk1 inhibition in tumor and neurodegeneration.

Taking together, our experiments have identified that the Chk1-CIP2A-PP2A pathway plays a key role in mediating AD-tau pathology and amyloidosis. However, other mechanisms, such as a direct phosphorylation of tau/APP by Chk1, and involvement of downstream targets of Chk1 other than CIP2A, may also participate in DNA damage–promoted AD pathogenesis, which needs further exploration.

In conclusion, we have discovered that DNA damage–induced Chk1 activation promotes tau and APP hyperphosphorylation and cognitive dysfunction in AD, and the Chk1-CIP2A-PP2A pathway may be involved in mediating this pathogenesis. Inhibition of Chk1 effectively reverses AD-like pathological changes. More and more studies have indicated shared molecular mechanisms between cancer and AD. Our current study reveals a new one, and points to the possibility of AD treatment by Chk1 inhibitors. Although the effect of antitumor drugs on neurodegenerative disease treatment is promising, further investigations are still needed to clarify the clinical relevance of these anticancer drugs to the treatment of neurodegenerative disease.

## Supplementary Information

Below is the link to the electronic supplementary material.

Supplementary file1 (DOCX 850 KB)

Supplementary file2 (XLSX 463 KB)

Supplementary file3 (PDF 516 KB)

Supplementary file4 (PDF 516 KB)

Supplementary file5 (PDF 516 KB)

Supplementary file6 (PDF 516 KB)

Supplementary file7 (PDF 516 KB)

Supplementary file8 (PDF 516 KB)

Supplementary file9 (PDF 516 KB)

Supplementary file10 (PDF 516 KB)

Supplementary file11 (PDF 524 KB)

Supplementary file12 (PDF 516 KB)

Supplementary file13 (PDF 516 KB)

Supplementary file14 (PDF 516 KB)

Supplementary file15 (PDF 516 KB)

Supplementary file16 (PDF 516 KB)

Supplementary file17 (PDF 516 KB)

Supplementary file18 (PDF 516 KB)
