# Supplementary material for: Chk1 Inhibition Ameliorates Alzheimer’s Disease Pathogenesis and Cognitive Dysfunction Through CIP2A/PP2A Signaling
Source: Neurotherapeutics. 2022 Mar 14;19(2):570–91. doi: 10.1007/s13311-022-01204-z (PMC9226264; doi:10.1007/s13311-022-01204-z)
Supplement: Supplementary file 1 — Supplementary file1 (DOCX 850 KB) [file 13311_2022_1204_MOESM1_ESM.docx]

**Supplementary Figures**

**
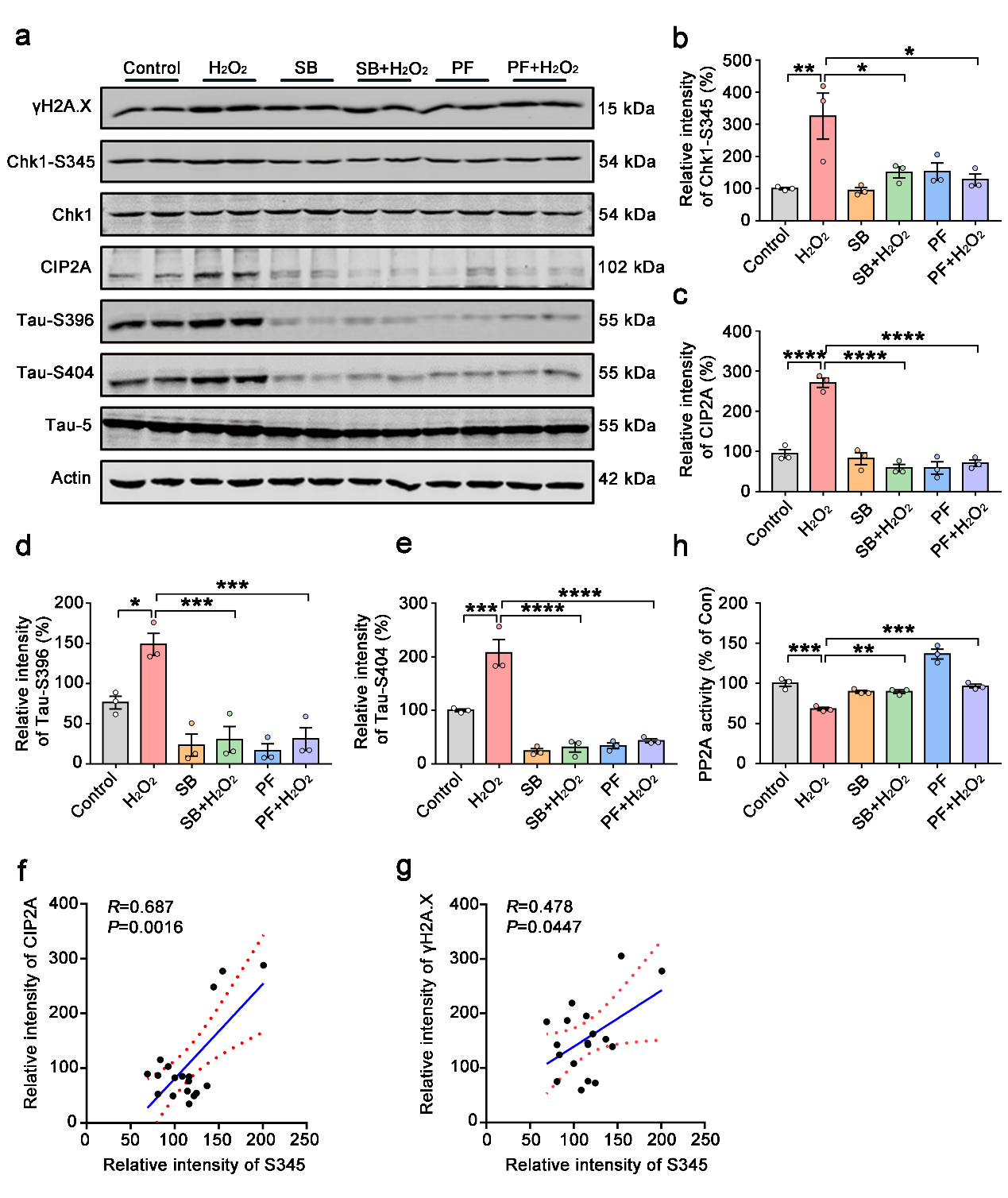
**

**Fig. S1 Chk1 inhibition reverses CIP2A overexpression and tau hyperphosphorylation caused by** **H_2_O_2_ in primary neurons**

(a-g) Primary neurons were treated with 200 µM H_2_O_2_ for 2 hours, with or without pre-incubation of ChK1 inhibitor SB218078 (1 μM) or PF477736 (1 μM) for 48 hours.

(a) Representative immunoblots of γH2A.X, Chk1-S345, Chk1, CIP2A, Tau-S396, Tau-S404, Tau-5 and β-actin.

(b-e) The quantitative analysis of the protein levels of Chk1-S345, CIP2A, Tau-S396 and Tau-S404 in (a).

(f) Correlation analysis of Chk1-S345 and CIP2A in (a)

(g) Correlation analysis of Chk1-S345 and γH2A.X in (a).

(h) PP2A activity assay. All data represent mean ± SEM, n=3, **P* <0.05, ***P* < 0.01, ****P*＜0.001, *****P*＜0.0001, comparison between the indicated two groups.

**Fig. S2 Chk1 inhibitor (GDC-0575) reduces tau phosphorylation and APP levels in cortex of APP/PS1 mice.**

**
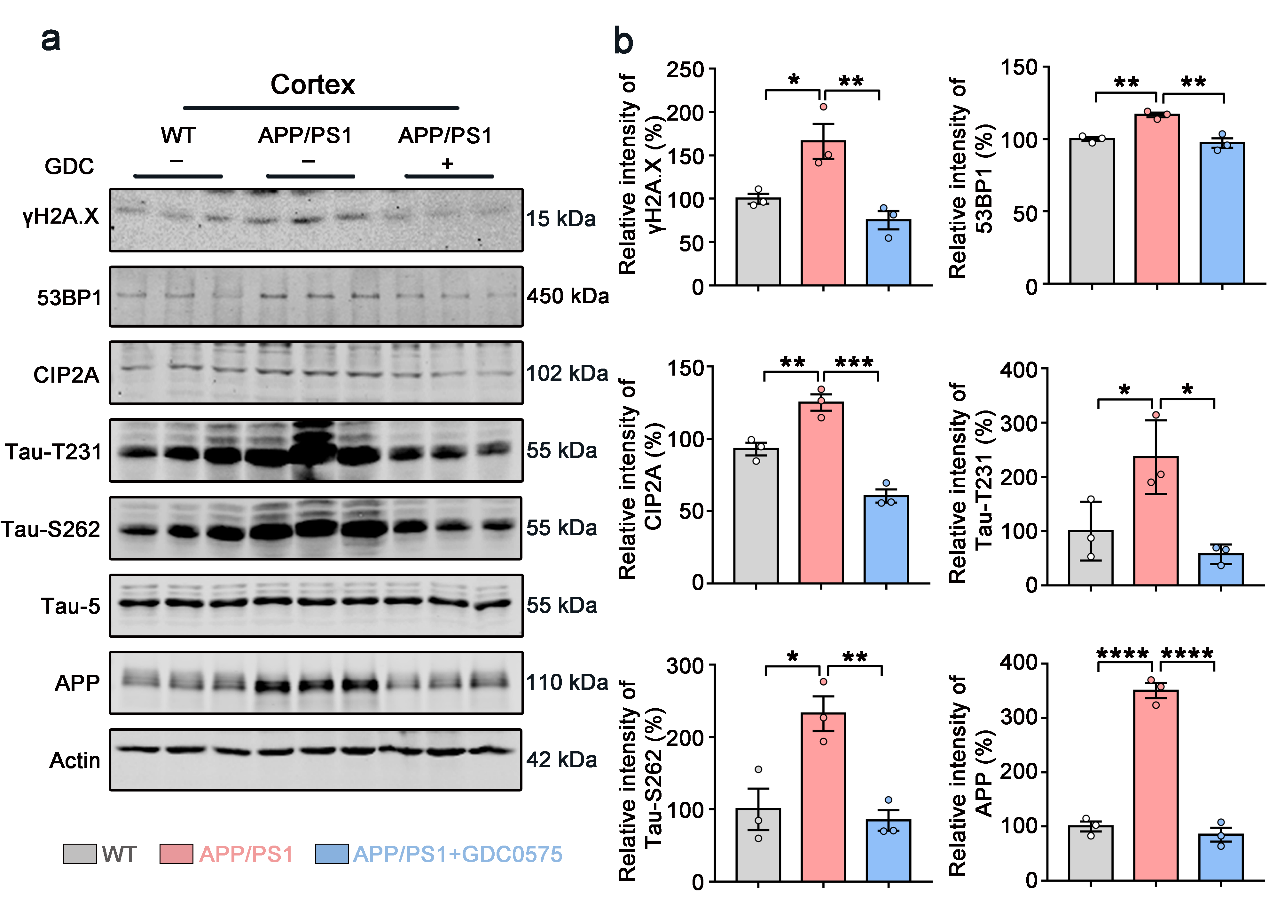
**

(a) Representative immunoblots of γH2A.X, 53BP1, CIP2A, Tau-T231, Tau-S262, Tau-5, APP and β-actin in cortex of the mice.

(b) The quantitative analysis of the protein level of γH2A.X, 53BP1, CIP2A, Tau-T231, Tau-S262 and APP in (a). All data represent mean ± SEM, n=3 per group, **P* <0.05, ***P* < 0.01, ****P*＜0.001, *****P*＜0.0001, comparison between the indicated two groups.
